# Cesium Lead Bromide-Coated Fiber Bragg Grating Sensors for Gamma Radiation Environments

**DOI:** 10.1021/acsomega.5c03281

**Published:** 2025-07-07

**Authors:** Tahira Khan, Manas R. Gartia, Jianwei Wang, Jyotsna Sharma

**Affiliations:** † Department of Petroleum Engineering, 5779 Louisiana State University, Baton Rouge, Louisiana 70803, United States; § Department of Mechanical and Industrial Engineering, 5779 Louisiana State University, Baton Rouge, Louisiana 70803, United States; ⊥ Department of Geology and Geophysics, 5779 Louisiana State University, Baton Rouge, Louisiana 70803, United States; # Center for Computation and Technology, 5779 Louisiana State University, Baton Rouge, Louisiana 70803, United States

## Abstract

This study presents the development and characterization of CsPbBr_3_ (CPB) and CPB-poly­(methyl methacrylate) (CPB-PMMA) composite coatings on Fiber Bragg Grating (FBGs)-based sensors for high-sensitivity gamma radiation sensing. CPB precipitates were synthesized using a solvent-based method and uniformly deposited onto the FBGs. The incorporation of PMMA into the CPB matrix enhanced both mechanical stability and adhesion to the FBG surface. Spectral analysis revealed significant Bragg wavelength shifts in response to gamma radiation exposures, indicating strain-optic variations induced by radiation–matter interactions. Comparative investigations between uncoated, CPB-coated, and CPB-PMMA-coated FBGs confirmed that the coatings significantly enhance strain sensitivity and stability. The incorporation of PMMA modified the mechanical response, influencing residual stress and strain attenuation. The advantage of using fiber optic sensors includes the ability to enable multiplexed sensing across large areas, immunity to electromagnetic interference (EMI), and operability in high-temperature environments. Additionally, CPB and CPB-PMMA coatings demonstrated enhanced sensitivity under UV exposure, further highlighting their potential for advanced optical sensing applications in both radiation and UV-intensive environments. These results demonstrate the potential of CPB-based coatings for radiation monitoring in extreme environments, including in nuclear facilities, space missions, and near medical instruments using radiation sources. The findings provide a foundation for further optimization of perovskite-polymer composites to enhance sensor performance and long-term durability in radiation-intensive applications.

## Graphical abstract

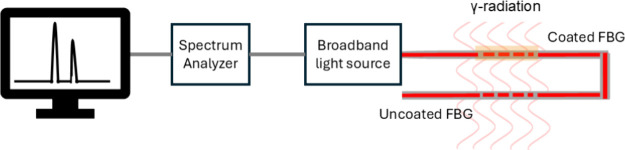

## 1 Introduction

Radiation detection devices are essential tools for identifying and measuring ionizing radiation, which can include alpha, beta, gamma, and neutron radiation. Gamma-radiation, due to its high energy and penetrating power, requires specific detection techniques to accurately assess its presence and intensity. Common methods for gamma radiation detection include scintillation detectors, high-purity germanium (HPGe) detectors, ionization chambers, semiconductor detectors, and solid-state detectors.^1–13^ Each of these methods operates on different principles, ranging from the production of light in scintillation materials to the generation of electron–hole pairs in semiconductors. Depending on the specific application—whether it is for environmental monitoring, nuclear safety, or gamma spectroscopy—these detection techniques offer varying levels of sensitivity, resolution, and practicality, making them indispensable in fields like health physics, radiation protection, and nuclear research.

Optical fibers, when coupled with scintillators, present a promising approach for radiation detection.^14–16^ In these systems, the optical fiber acts as a light guide, transmitting the light produced by scintillation materials when γ-radiation interacts with them. Scintillators, such as sodium iodide (NaI) or cesium iodide (CsI), emit flashes of light when γ-rays are absorbed. The optical fiber collects this light and guides it to a photodetector or photomultiplier tube, where it is converted into an electrical signal, which can then be analyzed. This combination allows for flexible, lightweight, and efficient radiation detection, particularly in environments where traditional detectors may be too bulky or sensitive to interference. The use of optical fibers in conjunction with scintillators also enables remote sensing and higher spatial resolution, making them suitable for various applications in radiation monitoring.

Fiber Bragg grating (FBG) sensors are another promising technology for detecting radiation, including γ-rays, through changes in the fiber’s physical properties induced by radiation exposure.^17–20^ An FBG consists of a periodic variation in the refractive index of the optical fiber, which reflects specific wavelengths of light. When the fiber is exposed to gamma radiation, it can experience changes in temperature, strain, or the refractive index of the fiber material, leading to a shift in the reflected Bragg wavelength. By monitoring these shifts, one can infer the radiation dose. FBGs offer several advantages, including high sensitivity, immunity to electromagnetic interference, and the ability to perform distributed sensing along the fiber. However, experimental studies have shown challenges, including sensitivity to environmental factors such as temperature, background vibrations, and humidity, which can influence the shift in the reflected Bragg wavelength.^21,22^ Additionally, while FBGs can detect changes in radiation exposure, their ability to distinguish between different types of radiation (such as γ- versus neutron radiation) remains a limitation, requiring further research and development to improve their specificity and overall performance in radiation detection.^22–29^

Cesium-based halide compounds, such as CsPbBr_3_ (Cesium Lead Bromide) and CsI (Cesium Iodide), are widely studied for their exceptional optoelectronic and scintillation properties, making them highly suitable for applications in radiation detection, imaging, and photonic devices.^30–36^ CsPbBr_3_, a lead halide perovskite, crystallizes in an orthorhombic structure at room temperature, transitioning to a cubic phase at higher temperatures.^37^ This perovskite provides excellent photoluminescence, high quantum efficiency, and defect tolerance, making it a promising candidate for X-ray and γ-ray detection, as well as light-emitting devices.^30,32,38–40^ Its superior carrier mobility and long carrier diffusion length further enhance its performance in optoelectronics.^30^ On the other hand, CsI crystallizes in a body-centered cubic (BCC) structure, which contributes to its high density and uniform scintillation properties. CsI is known for its high light yield, fast response time, and strong stopping power for ionizing radiation due to its high atomic number. These structural advantages make CsI ideal for medical imaging, homeland security, and high-energy physics experiments. The selection of CsPbBr_3_ or CsI is thus driven by their distinct crystal structures and associated optical, electronic, and scintillation properties, making them highly suitable for advanced radiation detection and optoelectronic applications.

In this study, we explore an innovative approach that leverages the properties of both FBGs and CsPbBr_3_ perovskite by coating FBGs with CsPbBr_3_ and CsPbBr_3_–PMMA. FBGs are widely recognized for their sensitivity to environmental factors such as temperature and strain, making them useful in diverse sensing applications.^41^ However, in radiation environments, conventional FBG-based gamma radiation sensors often show initial Bragg wavelength shifts (BWS) caused by temperature changes resulting from high radiation doses.^19^ These temperature fluctuations can cause transient peaks in the sensor’s response, which stabilize once thermal equilibrium is reached, as reported in ref 28. Unlike traditional sensors that rely on temperature or refractive index-induced BWS, CsPbBr_3_ undergoes lattice parameter changes upon exposure to gamma radiation.^42^ This lattice alteration induces strain in the coatings, which then induces strain in the FBGs, leading to observable peaks in the sensor’s response even at relatively low radiation doses. Notably, such low doses are insufficient to cause significant changes to the observed BWS in FBGs without coatings, indicating that they are primarily due to the strain effects from the lattice changes in the coating materials. These methods offer a distinct advantage over conventional FBG-based gamma radiation sensors, as they enable gamma radiation detection without the confounding influence of temperature-induced BWS. By leveraging the strain effects resulting from lattice changes in CsPbBr_3_ and PMMA-added CsPbBr_3_ coatings, our approach provides a more direct and sensitive means of gamma radiation detection, even at low doses of 5 μCi of Cs-137. Furthermore, various materials with distinct sensitivities to gamma radiation and neutrons could be selectively coated onto FBGs, enabling the differentiation between these two types of radiation. By carefully choosing coating materials with specific interaction mechanisms—such as scintillation, lattice deformation, or ionization effects, FBG sensors could be tailored to selectively respond to gamma rays or neutrons. This approach enhances the sensor’s versatility, allowing for precise radiation detection and discrimination in complex radiation environments.

This manuscript presents an investigation into the effects of CsPbBr_3_ coatings on FBGs for enhanced gamma radiation sensing. We describe the methods used for coating FBGs with CsPbBr_3_ and CsI, detailing the deposition process and the resulting structural modifications. The study explores how these coatings influence thermal and strain-optic variations in FBGs upon exposure to ultraviolet (UV) and gamma radiation, leveraging material-induced lattice changes to enhance sensitivity. To assess the impact of the coatings, we conducted a comparative analysis under UV and gamma radiation, examining the responses of bare FBGs, CsI-coated FBGs, and CsPbBr_3_-coated FBGs. CsI and CsPbBr_3_ were selected for comparison due to their fundamentally different radiation interaction mechanisms. CsI is a traditional scintillator that emits photons upon gamma exposure, potentially affecting the FBG through indirect optical pathways. However, under our experimental conditions, the CsI-coated FBG did not exhibit a significant Bragg wavelength shift, indicating that the scintillation light was insufficient to induce a measurable optical effect in the fiber sensor. In contrast, CsPbBr_3_ undergoes lattice distortions under gamma radiation, producing mechanical strain that directly modulates the Bragg wavelength of the FBG. This strain-based interaction enables more direct and sensitive detection. Our results demonstrate that CsPbBr_3_-coated FBGs show a pronounced strain-optic response even at low radiation doses, highlighting their superior performance in weak radiation fields. The results highlight the potential of these coated FBGs for radiation sensing applications, particularly in distinguishing between different radiation sources based on material interactions.

## 2 Experimental Section

### 2.1 Material Synthesis

The formation of the CsPbBr_3_ film was carried out in two steps.

In the first step, CsPbBr_3_ precipitates were prepared using the method described in the ref 36. Cesium bromide (CsBr) and lead bromide (PbBr_2_) were procured from a commercial vendor (Sigma-Aldrich) and used without further purification. A precursor solution of CsBr and PbBr_2_ was prepared in a 1.1:1 ratio by dissolving them in DMSO at 45 °C in a vial, followed by filtration. The solution was then cooled to 25 °C, after which acetone was added in small increments of 5 mL while stirring continuously. This process led to the formation of CsPbBr_3_ precipitates. Once stirring was stopped, the precipitates settled at the bottom of the vial, and the supernatant was immediately removed using a pipet. The process of adding acetone and removing the solution from the top was repeated several times until no DMSO remained in the vial. The materials have been fully characterized and the structural and physical analysis of CPB precipitates and undoped and PMMA-doped CPB films has been presented in a previous report.^36^

CsI was obtained from Sigma-Aldrich and was used without further purification. CsI was powdered further in agate mortar and pestal. 0.5 g of CsI was added in 5 mL predissolved mixture of acetone and PMMA and was stirred for few hours.

### 2.2 Coating Procedure

To form a film on the FBG, the precipitates, dispersed in acetone, were dripped onto a vertically positioned FBG and dried horizontally at room temperature. In a separate vial, a solution was prepared by dissolving 0.5 g of PMMA (poly­(methyl methacrylate)) in 5 mL of acetone at 30 °C. A small amount (1 mL) of this PMMA solution was added to the CsPbBr_3_ precipitates and stirred for 1 h. The resulting PMMA-CsPbBr_3_ mixture was then dripped onto an FBG and allowed to dry horizontally at room temperature. A similar approach was used to coat FBG with CsI-PMMA.

Figure 1a shows a comparison between an uncoated FBG and a CPB-coated FBG. The CPB-coated FBG remains relatively straight after drying, indicating uniform deposition with minimal induced stress. In contrast, Figure 1b presents an FBG coated with a composite mixture of CPB and PMMA. The addition of PMMA causes the fiber to curl upon drying, which can be attributed to the mismatch in the coefficients of thermal expansion (CTE) between the optical fiber and the polymeric coating. This differential shrinkage leads to internal residual stresses, resulting in the observed curling effect. The central wavelength of the CPB coated was originally 1553 nm and did not change after coating CPB film while the central wavelength of CPB-PMMA-coated FBG used in this study changed from 1553 to 1551 nm after coating CPB-PMMA film. While the central wavelength of uncoated FBG used in Figure 4 is 1550 nm.

**Figure 1:**
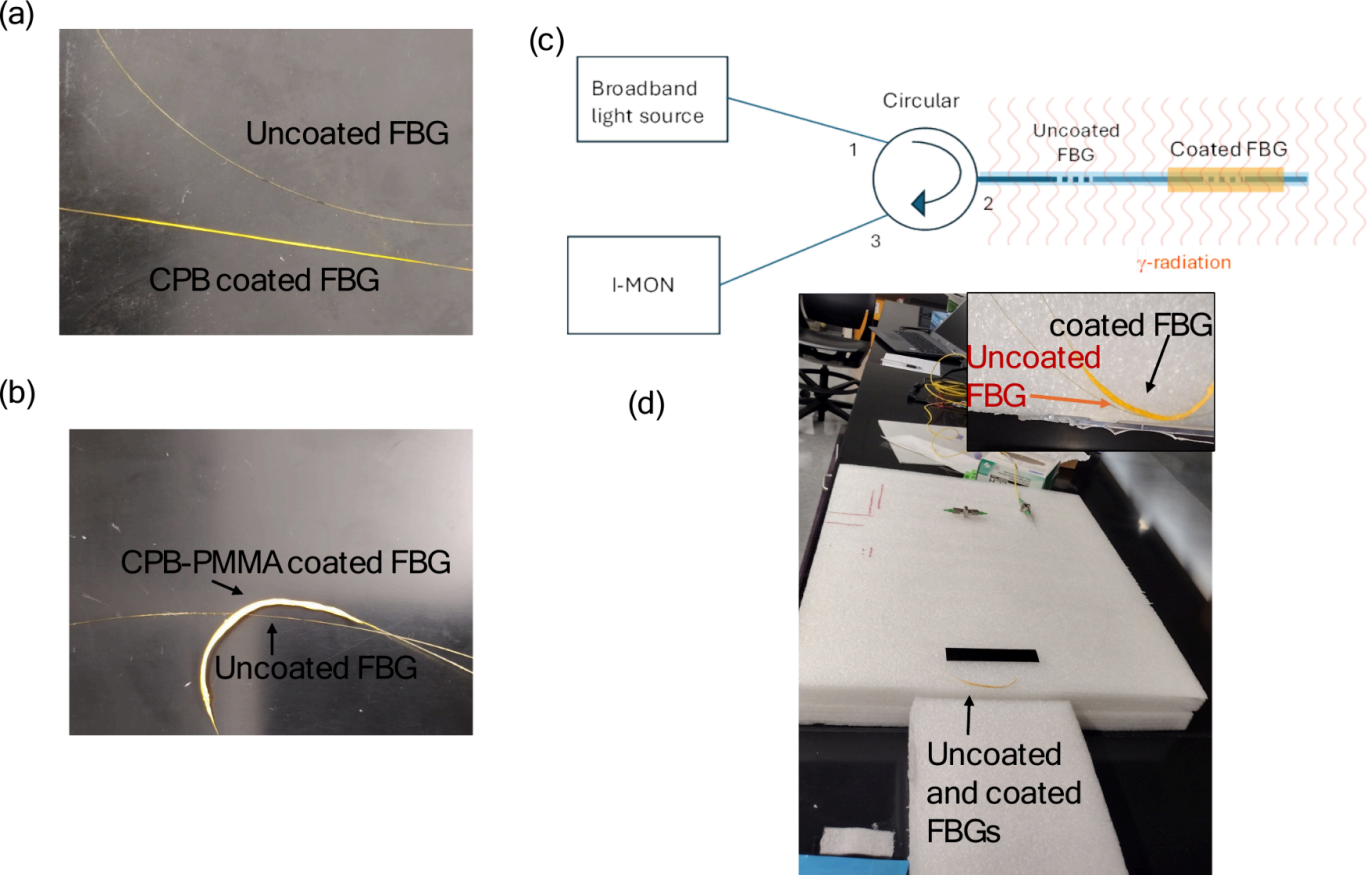
(a) Uncoated and CPB-coated FBG and (b) uncoated and CPB-PMMA-coated FBG (c) block diagram and (d) experimental setup for detection of γ-radiation where the inset shows the arrangement of coated and uncoated FBGs for exposure to gamma radiation. (“Photographs courtesy of ‘Tahira Khan’ Copyright 2025.”)

The experimental setup for gamma radiation exposure and spectral interrogation of the FBGs is schematically represented in Figure 1c. The coated FBGs were placed in a controlled radiation environment where they were exposed to gamma rays. The optical signals were injected into the fiber using broadband light sources, and the reflected Bragg wavelengths were continuously monitored using an optical spectrum analyzer. The interaction of gamma radiation with the CsPbBr_3_ coating was expected to induce structural and refractive index changes, which would be reflected in the BWS.

Figure 1d shows the actual experimental setup, where the coated FBGs were securely mounted on a structured foam platform to ensure proper alignment and mechanical stability. The optical fibers were connected to interrogation equipment for real-time spectral analysis. The experiment was designed to study the impact of gamma radiation on the CPB-coated FBGs, investigating potential variations in strain, refractive index, and mechanical integrity of the fiber due to radiation-induced effects. Cs-137 of 5 μCi was used as a γ-radiation source.

At a constant temperature, the Bragg wave shift (BWS) is related to the strain by the following relation.^43^

$$\frac{\Delta \lambda }{\lambda_{B}}=(1-P_{e})\varepsilon$$ 1

Where *P*_e_ is photoelastic constant which is 0.22 at room temperature and ε is strain. Δλ is BWS and λ_B_ Bragg wavelength (central wavelength of FBG).

### 2.3 FBG

Fiber Bragg Gratings were used as the core sensing elements in the system. These optical sensors reflect specific wavelengths of light while transmitting others, allowing them to detect variations in strain and temperature along the fiber. When external conditions such as stress or heat cause a change in the grating’s spacing, the reflected wavelength shifts accordingly. This wavelength shift is then analyzed to determine the physical changes the FBG has experienced. Their compact size, immunity to electromagnetic interference, and multiplexing capability make them ideal for sensing in harsh environments.

### 2.4 Interrogator and Light Source

The FBG sensing system was illuminated using a DL-BP1-1501A broadband light source. This unit is based on a superluminescent LED (SLED) design, offering a wide and stable spectral output around the telecom wavelength band. Integrated with an optical circulator, the DL-BP1-1501A enables efficient delivery of broadband light to the FBGs while routing the reflected signals toward the detection system.

For signal interrogation, an I-MON 512 USB optical spectrum interrogator was used. This compact, USB-powered device tracks shifts in the reflected wavelengths from the FBGs in real time. Its fast acquisition rate and high resolution make it well-suited for dynamic sensing scenarios.2.5 Light and Gamma Sources.

### 2.5 Gamma and UV Sources

To evaluate the FBG sensors under radiation exposure, both gamma and ultraviolet sources were utilized. A Cesium-137 (Cs-137) gamma source with an activity of 5 microcuries (μCi) was used to expose the sensors to ionizing radiation. A UV light source with an output power of 100 mW was employed to investigate the effects of high-energy ultraviolet exposure on the FBG sensors.

## 3 Results and Discussion

### 3.1 Coating Performance

The synthesized coatings were evaluated for their effectiveness in radiation shielding and long-term stability. The uniformity, adhesion, and interaction of the coatings with FBG sensors were systematically assessed by spectral analysis. Experimental observations confirmed strong adhesion, as evidenced by the central wavelength shift and intensity variations shown in Figure 2b. The central wavelength of the reference (uncoated) FBG is 1558 nm, while the uncoated FBG had a central wavelength of 1550 nm before coating. After coating with a CPB-PMMA film, the central wavelength shifted to 1549 nm, and subsequent coating of PMMA on top of the CPB film further shifted it to 1547 nm. This phenomenon, where the application of coatings alters the central wavelength of FBGs, has been observed in various studies.^44,45^ Their strong adhesion characteristics ensure consistent application onto FBGs, which is crucial for reliable sensor performance. The successful deposition of CPB-PMMA layer was validated through spectral analysis. Additionally, Figure 2c compares the Bragg wave shift (BWS) of a reference FBG and an uncoated FBG, while Figure 2d presents the strain response calculated from the BWS data using eq 1. These coatings not only improved strain sensitivity but also enhanced the stability of FBGs.

**Figure 2:**
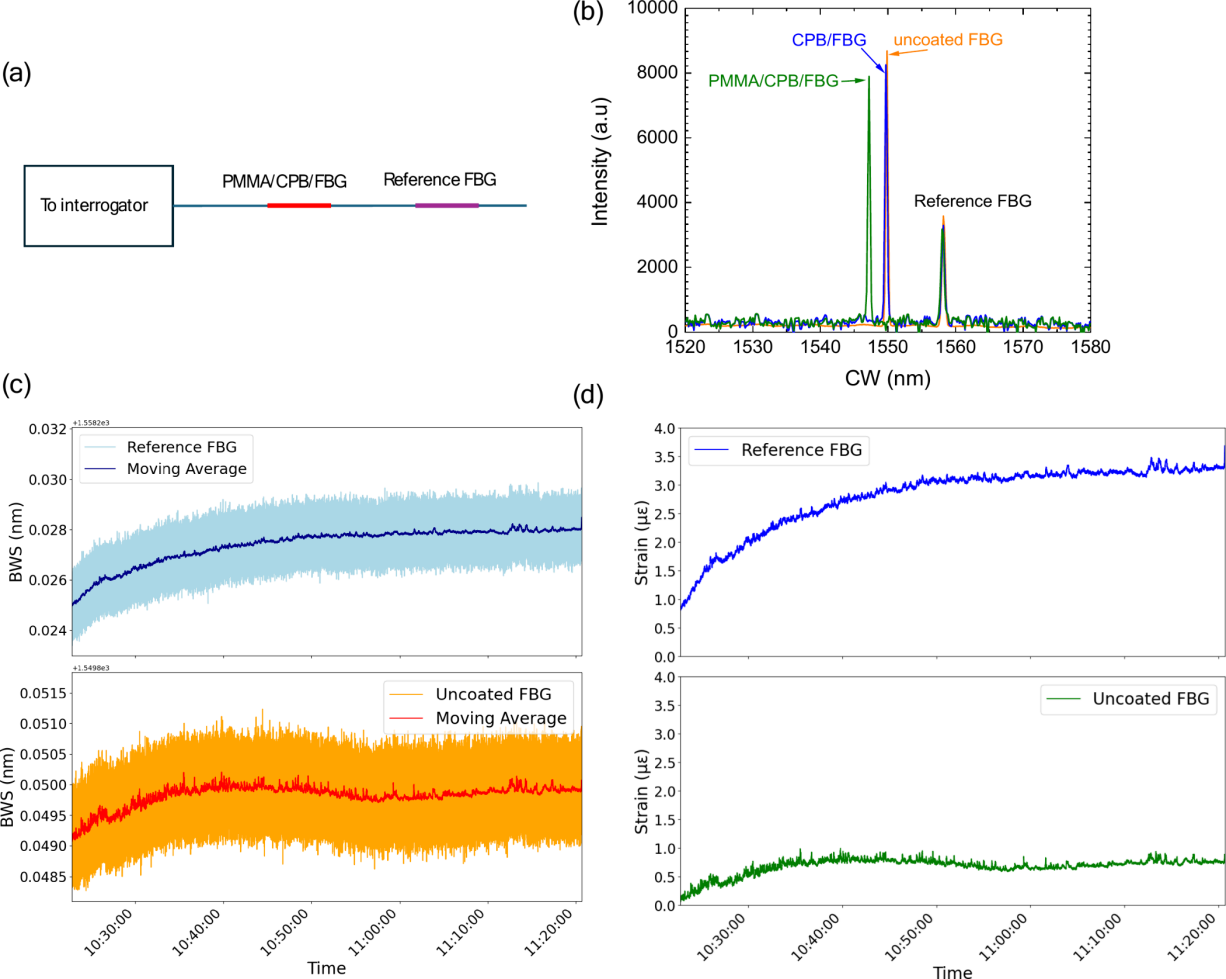
(a) Block diagram for experimental setup and (b) shows how the central wavelength and intensity of the peak is changing with coatings on the FBG. (c) and (d) are BWS and strain calculated from BWS where 1 με = 1 × 10^−6^ ε.

### 3.3 FBG Response under UV

Figure 3 presents the response of coated and uncoated FBGs under UV exposure. The central wavelength of the CPB-PMMA-coated FBG was initially 1553 nm and shifted to 1551.89 nm after coating. In contrast, the uncoated FBG used in Figure 3a had a central wavelength of 1550 nm. Similarly, the central wavelength of the CsI-PMMA-coated FBG was originally 1559 nm and shifted to 1558 nm after coating. Meanwhile, the uncoated FBG used in Figure 3b exhibited a central wavelength of 1547 nm. In Figure 3a, the CPB-PMMA-coated FBG exhibits significantly higher strain variations compared to the uncoated FBG, with distinct peaks corresponding to UV exposure periods (shaded regions). The inset image confirms the experimental setup used for testing. Similarly, in Figure 3b, the CsI-PMMA-coated FBG shows a pronounced strain response under UV exposure, while the uncoated FBG exhibits minimal variation.

**Figure 3:**
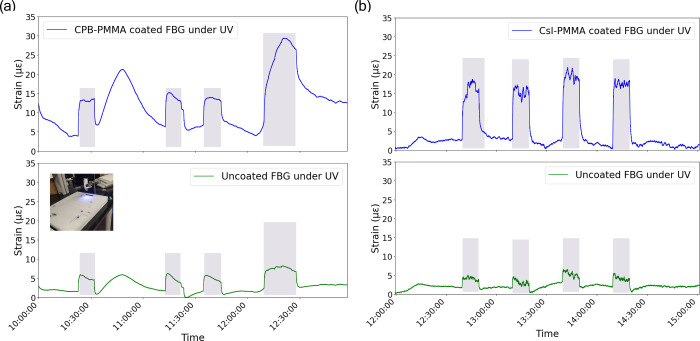
(a) Strain for CPB-PMMA-coated and uncoated FBG and (b) for CsI-PMMA-coated and uncoated FBGs under UV. The inset in (a) shows the experimental setup. The shaded area corresponds to the exposure to UV while the rest of the peaks are due to environmental effects. (“Photograph courtesy of ‘Tahira Khan. Copyright 2025.”)

A closer analysis reveals that the CsI-PMMA-coated FBG exhibited a more prominent strain response under UV radiation compared to the CPB-PMMA-coated FBG. This is evidenced by sharper and more significant strain peaks during UV exposure, suggesting that CsI has a stronger effect in this context. While CPB, as a lead halide perovskite, typically exhibits strong photonic interactions, CsI appears to respond more effectively to UV light in this experimental setup, possibly due to its optical absorption properties under the given conditions. CsI may have a stronger interaction with UV photons, resulting in larger strain variations compared to CPB, even though CPB is traditionally known for its high sensitivity to light.

The observed strain variations are likely caused by photostrictive or thermally induced expansion effects in the polymer matrix and the embedded materials.^7,8^ The more significant response of CsI may indicate that, in certain setups or under specific UV wavelengths, it can outperform CPB in terms of sensitivity. These results suggest that the coating materials enhance the sensitivity of FBGs to UV radiation, making them promising materials for optical sensing applications.

### 3.4 FBG Response under Gamma Radiation

The strain response of coated and uncoated FBGs under γ-radiation is depicted in Figure 4. In Figure 4a, the CPB-coated FBG shows small but noticeable variations when exposed to γ-radiation (shaded regions). In contrast, the uncoated FBG exhibits a minor increase in strain over time. This suggests that the CPB coating provides sensitivity enhancement to gamma radiations. In Figure 4b, the CPB-PMMA-coated FBG exhibits a stronger and unique strain response compared to the uncoated FBG, with a well-defined significant decline after exposure periods. A key feature in the strain response is the initial peak rise upon γ-radiation exposure, followed by a subsequent decline under continued radiation. Some studies attribute such peak behavior to temperature variations under high-dose γ-radiation exposure of 5.3 kGy/h dose rate.^19^ However, in this work, a weak radiation source (5 μCi) was used, ruling out significant temperature effects. Instead, the observed strain evolution can be attributed to structural changes within the CPB coating. Specifically, CPB undergoes a change in its lattice parameter under radiation,^46^ leading to strain modifications in the coated FBGs. The higher strain in CPB-PMMA-coated FBGs suggests that the PMMA layer further amplifies these effects, possibly by enhancing mechanical interactions or stabilizing the CPB structure under γ-radiation exposure. The reduction of the peak after reaching saturation under γ-radiation exposure, observed in both uncoated and coated FBGs, suggests that a relaxation process takes place shortly after the radiation starts. The phenomenon is not solely due to the coatings but is an intrinsic response of the FBG itself which is also reported by others.^19,24^

**Figure 4:**
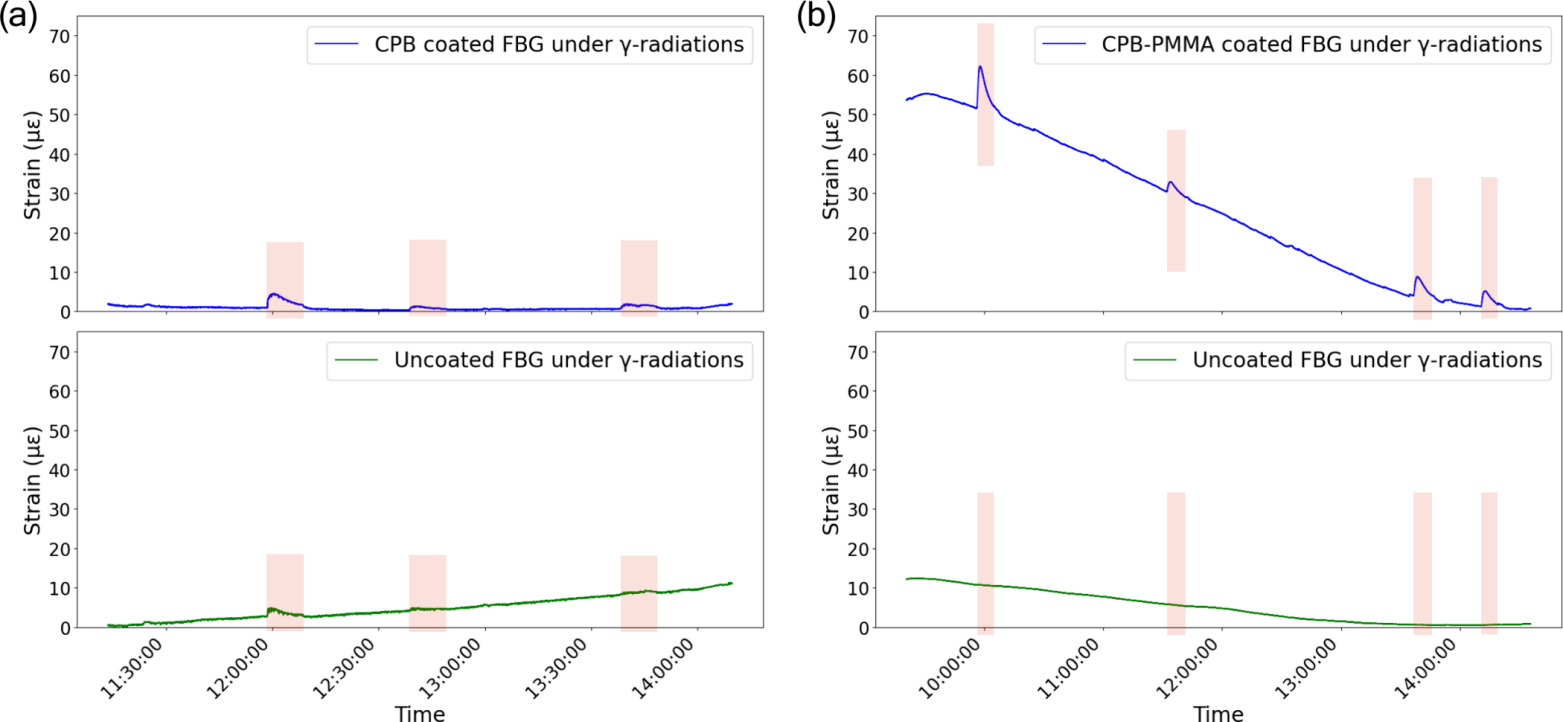
(a) Strain as a function of time for CPB-coated and uncoated FBG and (b) for CPB-PMMA-coated and uncoated FBGs under γ-radiations. The shaded area corresponds to the exposure to the γ-radiation.

The key factor contributing to the reduction of the peak after reaching saturation is the change in the refractive index of the fiber core.^17^ γ-radiation induces defects that modify the electronic structure of the silica matrix, leading to variations in the refractive index. This, in turn, affects the Bragg wavelength shift and influences the strain response of the FBG. Charge trapping and recombination within the fiber core and cladding can further contribute to these refractive index changes, leading to a decline in the strain response over time.^19,24^

Since this reduction is observed in both uncoated and coated FBGs, it indicates that while coatings like CPB and CPB-PMMA enhance sensitivity, they do not fundamentally alter this intrinsic relaxation behavior of the fiber under radiation exposure. Instead, they may modulate the rate or magnitude of the effect by influencing mechanical coupling, stress distribution, and refractive index evolution. These findings highlight the critical role of CPB and CPB-PMMA coatings in modulating strain responses, making them valuable for radiation-sensing applications. One potential approach to mitigating this decline is the use of fluorine-doped (F-doped) fiber-based FBGs, which exhibit improved resistance to radiation-induced defects and refractive index changes, making them a promising alternative for enhanced radiation tolerance.

Figure 5 illustrates the strain response of CPB-coated and uncoated FBGs under γ-radiation exposure over time. Figure 5a–c depicts the strain evolution as a function of time for different conditions: (a) uncoated FBG, (b) CPB-coated FBG, and (c) CPB-PMMA-coated FBG. In each case, the strain response shows distinct variations between coated and uncoated samples, highlighting the impact of CPB and CPB-PMMA coatings on strain sensitivity under radiation exposure. The strain values for the uncoated FBG (Figure 5a) reach approximately 2.5 με, while the CPB-coated FBG (Figure 5b) exhibits a higher strain of 3.5 με, and the CPB-PMMA-coated FBG (Figure 5c) shows the highest strain response at 11 με, indicating a significant improvement due to the coatings. Figure 5d–f presents the normalized strain data corresponding to (a–c), allowing for a comparative analysis of the strain evolution across different coatings. Normalization facilitates a clearer understanding of the strain dynamics, revealing the coatings’ attenuation trends and potential stability under repeated radiation exposure.

**Figure 5:**
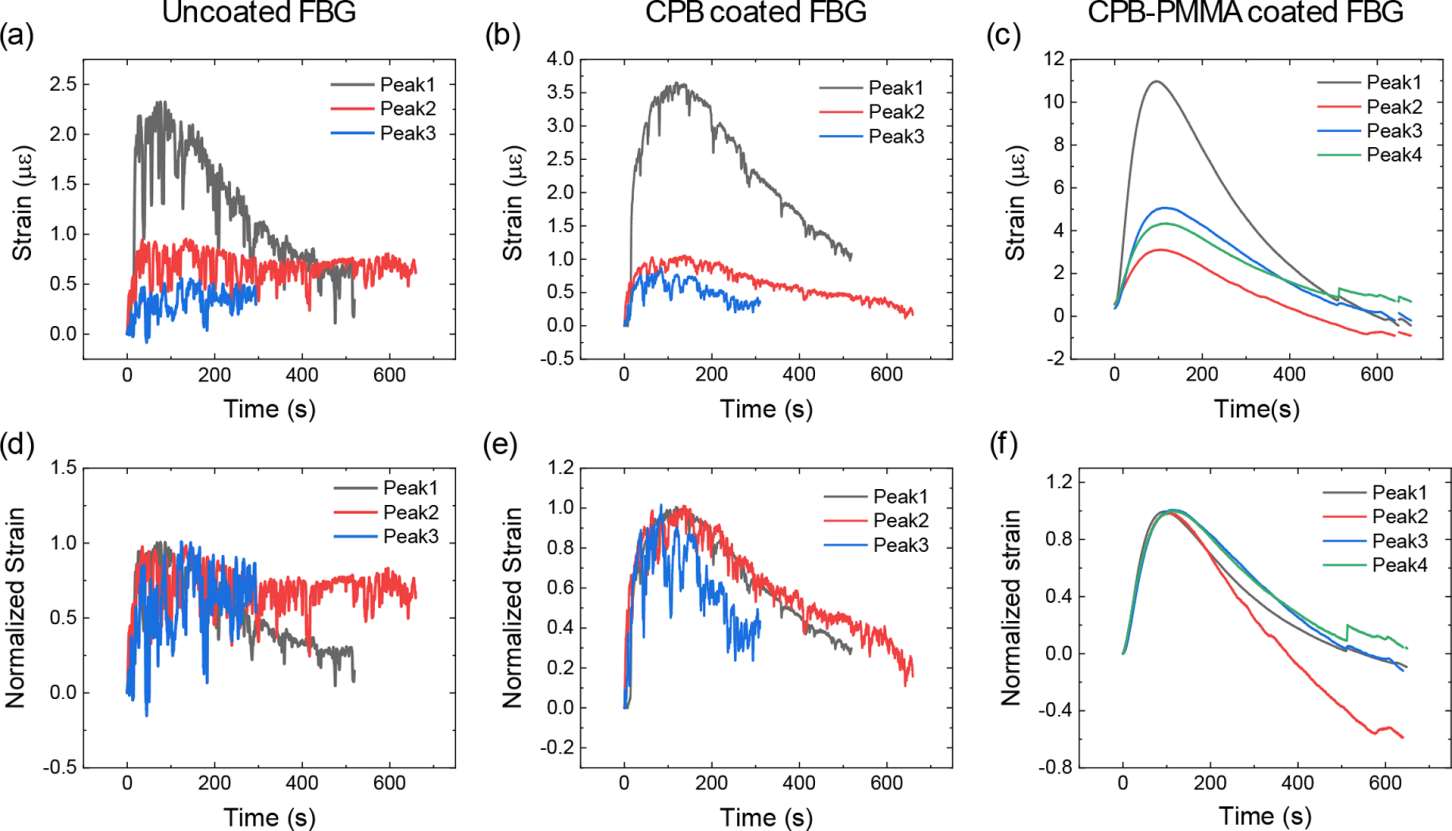
(a–c) depicts the strain evolution as a function of time for different conditions: (a) uncoated FBG, (b) CPB-coated FBG, and (c) CPB-PMMA-coated FBG and (d–f) presents the normalized strain data corresponding to (a–c), allowing for a comparative analysis of the strain evolution across different coatings. While the relative noise level for uncoated FBG is the highest, the absolute noise levels are similar among different measurements.

It is worth noting that the strain values in Figure 5c exhibit a transition into negative values, indicating compressive strain in the FBG. This effect likely results from stress relaxation dynamics in the CPB-PMMA coating, where lattice contraction or viscoelastic recovery in the polymer layer reverses the strain direction over time. Additionally, in Figure 5f, the normalized strain response during Peak 2 shows a deviation from the expected decay trend after approximately 200 s. This anomaly may be attributed to a combination of point source misalignment, coating nonuniformity, or residual stress effects from previous exposure. These factors can result in altered stress distribution and contribute to local deviations in sensor response.

The repeatability trends indicate that the CPB-PMMA-coated FBG shows a more stable and consistent response to gamma radiation exposure compared to the uncoated FBG. While there is some variability in peak intensity for the CPB-PMMA-coated FBG, attributed to factors such as point source misalignment and inconsistent time intervals between exposures, the coated sensor still demonstrates a clearer, more sustained signal over repeated exposure than the uncoated FBG, which shows more significant signal degradation. The diminished peaks observed in the CPB-PMMA-coated FBG during subsequent exposures are likely due to the point source not being perfectly aligned during the second exposure and the nonuniform time duration between exposures, which may have affected the sensor’s performance. Despite these factors, the CPB-PMMA coating shows a strong potential for reliable gamma radiation detection, maintaining its sensing capabilities over multiple exposures, whereas the uncoated FBG experiences a faster decline in performance. The observed differences between coated and uncoated samples suggest the role of CPB and CPB-PMMA coatings in modulating strain responses, which is critical for radiation-sensing applications.

The reduction of strain response can be modeled by two exponential functions, which represent the accumulation of radiation and strain and relaxation of the strain. This two-exponential model assumes that the strain response is governed by two primary, separable mechanisms: a rapid rise due to radiation-induced lattice expansion or deformation, and a slower decay due to relaxation processes such as viscoelastic behavior of the PMMA matrix or charge recombination. This approximation is appropriate for the low-dose regime used in this study, where strain effects remain linear and additive, and no permanent degradation or nonlinear response is observed. The saturation times for each of the peaks are similar, ∼90 s, with relaxation time ∼300 s. The peak strain response depends on each of measurements, which was not controlled.

The observed BWS is primarily attributed to strain induced by gamma-radiation-driven lattice expansion or distortion in the CPB coating. When embedded in a polymer matrix such as PMMA, this strain is more efficiently transferred to the fiber surface due to enhanced adhesion and mechanical coupling. γ-radiation may also produce localized electronic defects in both the CPB and the optical fiber, but the dominant measurable effect appears to be mechanical in origin. The CPB-PMMA-coated FBG thus acts as a composite sensing element where the perovskite serves as the active strain transducer and the PMMA layer acts as a stress-transfer interface.

In addition to the observed lattice parameter changes and refractive index modulation, it is also plausible that gamma radiation induces charge separation within the CPB and at the CPB/PMMA or CPB/fiber interfaces. Such charge dynamics may contribute to localized electric fields, which in turn could influence internal stress distributions and refractive index variations in the fiber core. While the current study focuses primarily on mechanical strain effects induced by structural distortions, the role of interfacial charge separation cannot be ruled out and warrants further investigation. Future work will consider techniques such as surface potential mapping and time-resolved photoconductivity to directly evaluate the contributions of charge accumulation and separation at these interfaces to the overall FBG response under radiation exposure.

### 3.5 Implications and Applications

The results of this study demonstrate the potential of CPB and specifically CPB-PMMA-coated FBGs for applications in radiation-intensive environments, with a key advantage being their distributed sensing capability. In our setup, two FBGs were used simultaneously, which can be extended to a larger array of FBGs capable of monitoring radiation over a broad area, in contrast to traditional point sensors.

The observed improvements in strain sensitivity and stability under gamma radiation exposure suggest that these coatings can be effectively employed in various fields requiring real-time structural health monitoring. In nuclear power plants, coated FBGs can be integrated into reactor components to detect radiation-induced strain variations, enabling predictive maintenance and enhancing safety. Similarly, in space missions, where exposure to high levels of gamma radiation is inevitable, the use of CPB-based coatings could improve the resilience of optical sensing systems, ensuring accurate structural monitoring of spacecraft materials over extended missions. Additionally, medical applications such as radiation therapy dosimetry could benefit from these sensors, providing precise real-time radiation exposure measurements. The use of these coatings in high-energy physics laboratories, where gamma radiation is frequently encountered, also holds promise for advanced monitoring systems in particle accelerators and other experimental setups.

To further optimize the performance of coated FBGs, several improvements can be considered. The development of enhanced polymer matrices with superior radiation shielding and mechanical stability could prolong sensor lifespan and maintain consistent performance. Additionally, optimizing the thickness of CPB-PMMA coatings could lead to better control of strain sensitivity, ensuring a more tailored response in different application scenarios. Another area of future research is the investigation of multilayered coatings that combine different materials to maximize shielding efficiency and sensor durability. Long-term radiation exposure studies are also necessary to evaluate potential degradation effects and ensure sustained performance in extreme environments. These advancements will contribute to the broader adoption of FBG-based radiation sensors across critical industries.

While this study has demonstrated the capability of detecting radiation, it is important to acknowledge the limitations, such as the inability to directly measure radiation levels, which could be a focus for future research. Additionally, limited data on long-term effects and performance under varying conditions are areas that need further investigation. Addressing these limitations will contribute to the broader adoption of FBG-based radiation sensors across critical industries. While the present study demonstrates the effective use of CPB and CPB-PMMA coatings for real-time gamma sensing under low-dose conditions, it does not address long-term stability or cumulative radiation effects. A comparative study examining the structural integrity, optical properties, and sensing performance of CPB and CPB-PMMA coatings across a range of gamma doses would be valuable in understanding the dose-dependent behavior and potential degradation mechanisms. Future work will focus on these aspects to establish performance thresholds and optimize composite designs for sustained use in radiation-rich environments.

## 4 Conclusions

This study demonstrates, for the first time, that CPB combined with PMMA coatings significantly improves the performance of FBG sensors in gamma radiation environments. To the best of our knowledge, this is the first study to establish a stable radiation sensing capability using this specific combination. The strong adhesion and structural integrity of these coatings ensure reliable sensor operation, while their ability to modulate strain responses under radiation exposure highlights their potential for radiation-sensing applications. The experimental results confirmed that CPB-coated FBGs exhibit noticeable variations in strain, suggesting their role in either shielding or enhancing sensor sensitivity. Furthermore, the addition of PMMA to CPB resulted in an even stronger and repeatable strain response, indicating that the composite material effectively enhances the sensor’s ability to detect radiation-induced changes. Additionally, CsI and CPB-PMMA coatings demonstrated enhanced sensitivity under UV exposure, further highlighting their potential for advanced optical sensing applications in radiation and UV-intensive environments. These findings establish CPB-PMMA as a promising material for improving the functionality of FBG-based sensors in extreme conditions.

The impact of these coatings on FBG sensor performance suggests several directions for future research, particularly in enhancing distributed radiation sensing capabilities. The ability to deploy multiple FBGs along a single fiber allows for the simultaneous monitoring of radiation across a wide area, rather than being confined to a single point, which is a significant advantage over traditional sensors. This makes the combination of coated FBGs and optical fibers highly promising for large-scale radiation monitoring in critical environments. Expanding the range of perovskite-based materials and polymer composites could lead to further improvements in sensor efficiency, durability, and sensitivity, especially in distributed sensing setups. Additionally, optimizing these materials for multipoint radiation detection could provide more detailed insights into radiation exposure patterns and improve overall system reliability. Investigating the effects of prolonged radiation exposure, along with environmental factors such as temperature and humidity, will be essential to validate the long-term reliability of coated sensors. Additionally, integrating these sensors with wireless monitoring systems could enable remote, real-time data collection in nuclear power plants, space missions, and medical radiation facilities. Finally, field testing in practical environments will be necessary to bridge the gap between laboratory results and real-world applications. By addressing these challenges, the development of advanced coated FBG sensors will contribute to the next generation of radiation monitoring technologies.

This is the first demonstration of a γ-radiation sensor based on CPB-PMMA composite coatings on FBGs, where the detection mechanism is strain-optic modulation induced by radiation-triggered lattice deformation. The CPB acts as the active radiation-responsive layer, undergoing lattice changes that generate strain upon exposure. This strain is effectively transferred to the fiber via the PMMA interface, producing measurable BWS. The observed responses are thus a combined result of coating-induced strain and possible secondary refractive index changes within the fiber. These insights form the basis for further material and structural optimization to refine and enhance radiation detection using this platform.

## Supplementary Material



## Notes

Morphological comparison of CPB and CPB-PMMA films and CPB-PMMA coated FBG response under noisy environment (PDF)
