# Supplementary material for: Cesium Lead Bromide-Coated Fiber Bragg Grating Sensors for Gamma Radiation Environments
Source: ACS Omega. 2025 Jul 7;10(28):30886–95. doi: 10.1021/acsomega.5c03281 (PMC12290937; doi:10.1021/acsomega.5c03281)
Supplement: Supplementary file 1 [file ao5c03281_si_001.pdf]

Supporting Information

Cesium Lead Bromide-Coated Fiber Bragg Grating Sensors for Gamma Radiation Environments

Tahira Khan[†, *], Manas R. Gartia[§], Jianwei Wang[⊥, #], and Jyotsna Sharma[†*]

[†] Department of Petroleum Engineering, Louisiana State University, Baton Rouge, Louisiana 70803, United States
[§] Department of Mechanical and Industrial Engineering, Louisiana State University, Baton Rouge, Louisiana 70803, United States
[⊥] Department of Geology and Geophysics, Louisiana State University, Baton Rouge, Louisiana 70803, United States
[#] Center for Computation and Technology, Louisiana State University, Baton Rouge, Louisiana 70803, United States

*Corresponding author; email: jsharma@lsu.edu, tkhan@lsu.edu

S1. Morphological Comparison of CPB and CPB-PMMA Films (SEM Analysis) 3

Figure S1 SEM images of (a) CPB film and (b) CPB-PMMA film, deposited on flat substrates using the same method as used for FBG coatings. 3

Figure S2 Strain as a function of time for uncoated and CPB-PMMA coated FBG under gamma radiation. The red regions show when the FBGs are exposed to gamma radiation. 4

S1. Morphological Comparison of CPB and CPB-PMMA Films (SEM Analysis)

To assess the surface morphology of the $CsPbBr_3$-based coatings used on FBGs, scanning electron microscopy (SEM) was performed on standalone films of CPB and CPB-PMMA, prepared using the same deposition protocol as that used for FBG coatings. Since the geometry of the FBG prevents direct SEM imaging, these films provide insight into the structural features relevant to sensor performance.

Fig. S1 shows SEM images of the CPB film (S1a) and the CPB-PMMA film (S1b). The CPB film exhibits a polycrystalline structure with microcrystallites averaging 2–4 μm in size and sharp grain boundaries, suggesting rapid and uncontrolled grain growth. In contrast, the CPB-PMMA film displays a more homogeneous microstructure with smaller, more evenly distributed crystallites and reduced grain size. The addition of PMMA appears to hinder grain coalescence and promote dispersion, likely due to steric effects and limited ion mobility within the polymer matrix.

This morphological modification plays a key role in enhancing adhesion to the FBG surface and improving strain transfer characteristics under gamma radiation exposure, as discussed in the main manuscript. The finer grain structure of CPB-PMMA also correlates with its improved mechanical stability and repeatable strain response during sensor operation.

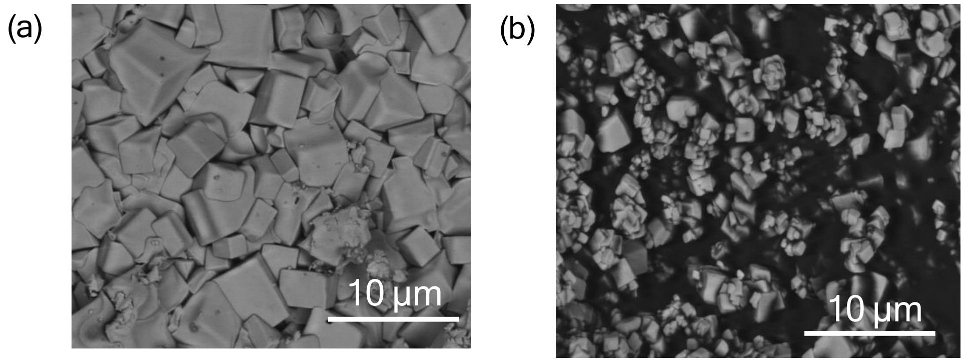

Fig. S1. SEM images of (a) CPB film and (b) CPB-PMMA film, deposited on flat substrates using the same method as used for FBG coatings. "Figure reproduced with permission from Tahira Khan, ACS Adv. Opt. Mater., Copyright 2025."

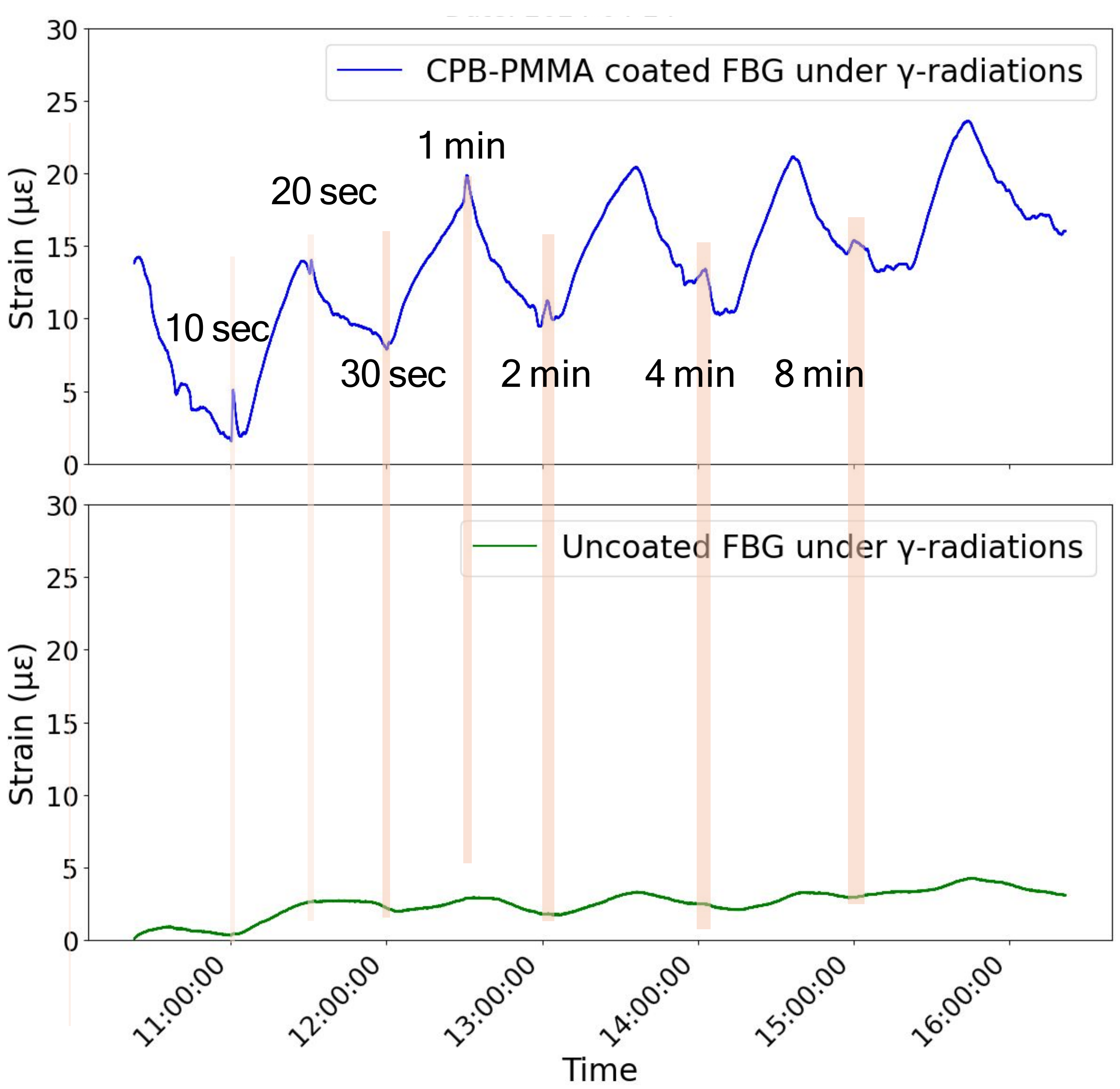

Figure S2 Strain as a function of time for uncoated and CPB-PMMA coated FBG under gamma radiation. The red regions show when the FBGs are exposed to gamma radiation**.**
